# CONCEPTUALIZING BLACK FEMINIST-WOMANIST GERONTOLOGY

**DOI:** 10.1093/geroni/igae098.3447

**Published:** 2024-12-31

**Authors:** Brandy Wallace, Cassandra Ford, Tamara Baker

**Affiliations:** University of Maryland Baltimore County, Baltimore, Maryland, United States; University of Alabama, Tuscaloosa, Alabama, United States; University of North Carolina, Chapel Hill, Chapel Hill, North Carolina, United States

## Abstract

There is a long history documenting Black women being particularly vulnerable to negative implicit and/or explicit behaviors, which marginalizes their social positioning and puts them at an increased risk of feeling powerlessness, and physically and emotionally stressed. This has created a chasm, particularly between this group and the healthcare industry - resulting in poorer health outcomes across generations of Black women. Recent scholarship has questioned the lack of culturally responsive, theory-guided research addressing the connections between aging, Black womanhood, and what is needed to advance health equity. Models that utilize traditional theories of aging often do not account for cultural context that undergirds the aging experience specifically among Black women. To understand the ways in which Black women thrive, we must consider various approaches that define their well-being. Dichotomizing aging into concrete categories as healthy/unhealthy may unintentionally isolate this group where aging successfully presents as a contradiction, thus perpetuating further marginalization. It is important that scholarship and intervention projects reflect cultural humility in dissemination. Applying Black-Feminist-Womanist methods in gerontology provides a curation of thought that creates a foundation by which Black women survive, live, and age, despite the ‘gold standard’ of aging being dominated by white ethnocentric context that pathologizes older Black women’s lived experiences. In this presentation, we summarize the principles and methodological approaches of Black Feminist and Womanist perspectives and use them to conceptualize “Black Feminist-Womanist Gerontology,” a culturally relevant model for older Black women. Factors of the model and recommendations of its use will be discussed.

